# Social integration and satisfaction in medical studies among foreign students in Tunisia

**DOI:** 10.1192/j.eurpsy.2025.1283

**Published:** 2025-08-26

**Authors:** N. Bouattour, F. Guermazi, M. Lalaoui Rhali, N. Regaieg, I. Baâti, J. Masmoudi

**Affiliations:** 1 Psychiatry A, Hedi Chaker University Hospital, Sfax, Tunisia

## Abstract

**Introduction:**

Foreign students are confronted to the multiple obstacles along their university studies. These obstacles can be related to the nature of the medical studies, the diversity and dense quantity of the subjects, and also to the interaction of the students with their colleges, their patients and the paramedical staff during the training. Psychological distress can, also affect the process of integration in society.

**Objectives:**

To assess the factors influencing the social integration and the satisfaction of the foreign students in the faculty of medicine in Sfax-Tunisia.

**Methods:**

We conducted a cross-sectional, descriptive and analytical study, carried out between July and September 2023 among foreign students at the Sfax Faculty of Medicine, via an anonymous self- questionnaire via “Google Forms shared via social media. Social integration was evaluated via the French version of the need to belong scale by Richer and Vallerand. The satisfaction of the medical studies was evaluated using the scale of satisfaction of studies by Vallerand and Bissonnette. Psychological distress was assessed using the “DASS-21” Depression, Anxiety and Stress Scale.

**Results:**

Seventy-two foreign medical students completed the survey. The average age was 25 ± 3.45 years. Males represented 57% of the total participants. The majority of the participants were north Africans 87.5% (Moroccan: 68.05%, Mauritanian: 18.05% and Algerian 1.4%). The majority of respondents were single (77.8%). Sixty-one percent were enrolled in the third cycle of medical studies, 26.5% were enrolled in the second cycle of medical studies and 12.5% were enrolled in the first cycle of medical studies. The average length of stay in Tunisia was 6.5 years. Seventy-seven percent were satisfied with the choice of Tunisia for the medical studies. The male gender was statistically associated to the feeling of a better social integration (p=0.045). In addition, social integration was statistically associated to satisfaction in medical studies (p=0.004, r=0.034) and to academic performance (p=0.008). We found no statistical association between the level of the students nor the length of the stay in Tunisia. We found no statistical association between social integration and psychological distress.

**Conclusions:**

The feeling of being an integral part of the social community can help alleviate the stress of adjusting to a new environment and improve emotional well-being, which can positively influence the learning experience and satisfaction of these foreign students.

**Disclosure of Interest:**

None Declared

